# Preparing for Care: A Clinician’s Perspective on Anticipatory Caregiving and Meaning Making

**DOI:** 10.2196/91508

**Published:** 2026-04-28

**Authors:** Vania Modesto-Lowe

**Affiliations:** 1 School of Health Sciences, Quinnipiac University, 370 Bassett Road, North Haven, CT, 06473, United States, 1 203-582-7782

**Keywords:** anticipatory caregiving, meaning making, caregiver burden, autonomy, partnership

## Abstract

More than 50 million adults in the United States provide unpaid care to a family member. Caregiving is associated with high rates of depression, anxiety, social isolation, financial strain, and physical exhaustion. Elevated caregiver distress has also been linked to poorer patient outcomes, including worse symptom control, lower adherence to treatment, and increased health care use. Despite this well-documented burden, most research and intervention efforts focus on caregivers once illness or disability is already advanced and strain is fully present. Far less attention has been given to anticipatory caregiving, the emotional and relational adjustments that begin before clear functional decline. As aging parents grow more frail, adult children often confront uncertainty, shifting roles, and the gradual loss of independence long before hands-on care is required. This period may be marked by vigilance, grief, and existential distress, yet it remains largely unaddressed in the literature. This perspective examines anticipatory caregiving through clinical reflection and personal narrative, highlighting the need to recognize and support caregivers earlier in the trajectory of aging, before crisis defines the experience.

As I prepare to see my mother in person after several months, I gather my passport and plan the obligatory family gifts. I also catch myself imagining how she will look. I find myself scanning ahead of time for signs of frailty. Will she walk with a limp that was not there before or need help with things she used to do on her own?

For many adult children, caregiving begins in this anticipatory space, long before hands-on care is required. Caregiving begins long before physical tasks and constitutes an extended psychological and relational process. From my perspective, that early phase is filled with vigilance, imagined loss, and a growing sense of responsibility for another’s vulnerability. These largely unspoken forms of emotional labor shape how we later engage in care. What I am describing is not only personal; it is well-documented in the literature.

In the United States, more than 50 million adults provide care to family members, many experiencing varying levels of distress. This distress can include physical fatigue as well as psychological effects, such as hopelessness, anticipatory grief, and loneliness [1]. Collectively, these experiences reflect caregiver burden: emotional, social, financial, physical, and spiritual strain [2]. High caregiver distress is associated with poor symptom management, reduced treatment adherence, and diminished patient quality of life. When caregivers are exhausted, their ability to sustain care, communicate with clinicians, and participate in decision-making falters [3].

Recognizing this, research, particularly in oncology, has focused on caregiver distress. Interventions offering education, skills training, and structured support have shown improvements in quality of life and reductions in anxiety and depression, especially when patients and caregivers participate together [1]. Still, findings are mixed. A 2019 Cochrane Review found that psychosocial interventions for informal cancer caregivers may yield small improvements in quality of life immediately post intervention, but likely have little to no sustained effect on depression, anxiety, or psychological distress. The authors noted substantial heterogeneity across studies and overall low methodological quality, limiting firm conclusions [4]. These findings do not suggest that caregiver support lacks value; rather, they may reflect brief, largely psychoeducational programs that were not individualized to caregivers’ specific needs, relational dynamics, or cultural context.

More recent meta-analyses offer nuance. Programs lasting 8 weeks or longer tend to reduce anxiety and depression. Meaning-centered approaches appear particularly helpful for the existential distress that caregivers often face [5]. In my clinical work, I often return to the questions at the heart of meaning-centered psychotherapy: Who am I in this role? What matters most to me? What do I want my loved one to feel from me?

These approaches emphasize the possibility of growth alongside burden. I recognize that process in myself, even before initiating any formal caregiving role.

I scan for changes: the way she steadies herself coming down the stairs. What I have not done is tell her I am scanning. My stomach tightens just thinking about it, so I stay quiet.

If I asked her, she would wave it off. “You cannot hide much from your own kid,” she would say. “But let’s not make a whole thing of it. I am fine.”

If I inquired about her daily routine, she would say, “I still cook everyone’s eggs in the morning, the way I have always done it. What else do you want?”

What does she want as she ages? Not surveillance. Not daughters ruling on her affairs. “I just want things to stay as normal as possible. I do not need fussing. I raised my family. I have completed my mission. That is enough. Just let me be.”

I tell myself I am monitoring, watching for signs, preparing.

But partnership is different from monitoring.

Partnership requires that I ask and then tolerate the answer. If she says she is fine, partnership asks me to sit with that, not to translate it into what I think she means, not to secretly catalog it as denial. Sometimes I want to steer the conversation toward the future, toward plans and contingencies. Instead, I try to stay where she is, though it is hard to do so.

I speak of anticipatory grief as if it belongs to me. But she, too, is aging inside a body that surprises her. She, too, may be watching me, measuring the steadiness of my visits, the tone of my voice, the frequency of my calls.

Ours is not a relationship of effortless intimacy. It never has been. But it has always been honest. I have been preparing to care for her in some practical way. I have not prepared to sit beside her as two women who are both afraid.

I assume she is proud of me in some respects. But pride travels in both directions. Have I made room to admire the woman she is, not just the mother she was? Since I was 10 years old, it has been difficult to manage my mother. We are not women who cook side by side. We are not soulmates. We do not share a hobby. But we both love the beach.

We walk for hours, scanning the horizon, meeting each wave as it comes. The collaboration is the commentary: relatives, my friends, the world as she narrates it with wit and grievance. Advice arrives whether requested or not, all of it delivered with impeccable timing.

That space, sharp and familiar, is ours.

My vigilance is not only about decline. It is about advocacy. When caregiving comes, my role will not be just to report symptoms but also to translate her values. Medical histories begin with “What is the matter?” But what she needs is space for what matters. To her, autonomy, normalcy, and dignity matter more than surveillance. My task is not to overtake her narrative but to safeguard it.

I wonder whether her doctors are tracking only her heart and lab values or also noticing her social life. We would notice if she stopped going out. Meanwhile, we teach her to use a tablet, not to become her instructors, but to keep her in the thread of things. The look on her face when she hears the ping of a message tells me it matters.

What she needs are people who are not family, places that expect her. The prescription may not be a medication at all, but something that requires her to show up: a Pilates class or a standing lunch. These are not luxuries. They give her a reason to get dressed and go out.

Meaning making coexists with logistics. Loving an aging parent also means entering the administrative maze of prior authorizations, banking safeguards, medication lists, hearing checks, home repairs, and unexpected charges. All of it is work. Naming it permits caregivers to feel overwhelmed not only by worry but also by the coordination of aging demands [2].

As I prepare to see my mother, this perspective helps me frame my anticipatory worry more gently. What are my hopes for this visit? How are they shaped by my values? What do my fears reveal about my relationship with her?

As I reflect, something shifts. As much as I dread witnessing her possible decline, I recognize that I want to be there. If she needs help, I want to be one of the people helping her. It is frightening to imagine her growing more vulnerable, but there can be meaning for both of us in sharing that journey.
